# Functional status following pulmonary rehabilitation in people with interstitial lung disease: A systematic review and meta-analysis

**DOI:** 10.1177/14799731241255138

**Published:** 2024-10-23

**Authors:** Guilherme Rodrigues, Rute Santos, Rita Pinto, Ana Oliveira, Alda Marques

**Affiliations:** 1 Lab3R – Respiratory Research and Rehabilitation Laboratory, School of Health, 56062 University of Aveiro(ESSUA), Aveiro, Portugal; 2 iBiMED – Institute of Biomedicine, 56062 University of Aveiro, Aveiro, Portugal; 3 Polytechnic Institute of Coimbra, Coimbra Health School, Coimbra, Portugal; 4 School of Rehabilitation Science, McMaster University, Hamilton, ON, Canada; 5 West Park Healthcare Centre, Respiratory Medicine, Toronto, ON, Canada

**Keywords:** “Interstitial lung diseases”, Rehabilitation, “Functional capacity”, “Functional performance”

## Abstract

**Background:**

Individuals with interstitial lung disease (ILD) often experience worsening symptoms and activity avoidance. Limited data exists on outcome measures for assessing functional status (capacity and performance), as well as on the effectiveness of pulmonary rehabilitation (PR) in improving these outcomes in ILD.

**Aim:**

This review aimed to systematically assess the effects of PR on both functional capacity and performance in individuals with ILD.

**Methods:**

Randomised controlled trials involving pulmonary rehabilitation (PR) in adults with ILD, which included at least an exercise training component and education and/or psychosocial support, were included. Risk of bias and quality of evidence were assessed. Mean changes from baseline and standard deviations were retrieved for each group, and a random-effects model was applied.

**Results:**

Eight studies were included, mostly involving individuals with idiopathic pulmonary fibrosis (*n* = 5). PR duration ranged from 3 to 26 weeks. Seven studies used the 6MWT to evaluate functional capacity and one also used the 30-s STS. Two studies assessed functional performance, measuring time spent in moderate physical activity with the SenseWear Armband, number of steps per day with the same device, and energy expenditure in MET-min using the international physical activity questionnaire. PR improved functional capacity (6MWT-MD 45.82 m, 95%CI [26.14; 65.50], I^2^ = 71.54%, *p* < .001; 30-s STS- PR: 3.7 ± 2.6 reps; control group: −0.4 ± 2.5 reps, *p* < .001) compared to usual care. Only self-reported physical activity levels increased after PR (PR: 51.4 ± 57.7MET-min; control group: 20.9 ± 37.2MET-min, *p* = .03).

**Conclusion:**

PR is effective at improving functional capacity; however, functional performance is often overlooked, resulting in limited and inconclusive findings.

## Introduction

Individuals with interstitial lung disease (ILD) often experience worsening symptoms, leading to limitations in their ability to perform activities^1^ and participate in daily life, thus affecting their functional status. Functional status, recently identified as a research priority in ILD,^2^ encompasses two main yet complementary concepts: functional capacity and functional performance.^3^ Functional capacity refers to a person’s maximum potential to perform a functional activity (e.g., walking, sitting, standing, or stair climbing) under controlled, standardised conditions, and it is typically measured through functional tests.^3^ Functional performance refers to what people actually do in the context of their daily lives and is mostly assessed through patient-reported outcome measures (PROMs), which may evaluate whether patients can perform activities of daily living or assess the impact of specific barriers to activity (such as dyspnoea).^4^ Quality of life measures, which can include sub-domains related to activities, may also be used; however, the data they provide serve as proxies for functional performance.^4^ Physical activity (PA) is also considered functional performance because it involves the volume and intensity of activities people engage in during their daily lives.^3^ Its assessment may be conducted with PROMs or objective measurements, such as activity monitors.^5^ Functional status is, therefore, a complex construct that is highly meaningful to patients and can potentially be improved through non-pharmacological interventions. Pulmonary rehabilitation (PR) is a non-pharmacological intervention that has been found to be safe and effective for individuals with ILD^6,8^; however, adherence to PR standards, which require exercise training and educational/psychosocial support as minimum components,^7^ varies among studies. Published systematic reviews^6,8^ have provided evidence from studies that adhere to the definition of PR; however, they also include results from exercise-only interventions. Combining data from these studies without recognising key differences hampers our understanding of the true impact of PR in several domains, including functional status in ILD. Moreover, there is limited data on the outcome measures used to assess functional capacity and performance in ILD, as no review has focused on summarizing these health domains. Hence, this review aimed to systematically assess the effects of PR on both functional capacity and performance in adults with ILD.

## Methods

This systematic literature review was reported according to the Preferred Reporting Items for Systematic Reviews and Meta-Analyses (PRISMA) statement^9^ (Section A1). The review protocol was registered at the International Prospective Register of Systematic Reviews (CRD42022298584).

### Eligibility criteria

Studies were included if they: i) were randomised controlled trials (RCTs); ii) included individuals aged ≥18 years with a clinical diagnosis of ILD; iii) implemented PR programmes including, at least, an exercise training component (e.g., strength and/or endurance training) and education and/or psychosocial support; iv) compared PR to usual care or any other intervention; v) reported outcome measures of functional capacity and/or performance and vi) were written in Portuguese, English, French or Spanish. Although not explicitly outlined in our registration protocol, RCTs with alternative models of PR delivery (e.g., home-based, telerehabilitation) were included if they involved supervised sessions; otherwise, they were excluded. Qualitative studies, grey/unpublished literature and studies involving mixed chronic respiratory diseases or non-traditional exercise modalities (e.g., yoga, tai chi, qigong) were excluded.

### Search strategy

The search was first conducted in December 2022 and rerun in March 2024 in PubMed/MEDLINE, Scopus, and Web of Science Core Collection. The complete search strategies for all databases can be found in the supplementary material (Section A1). CINAHL and EMBASE were not searched as originally planned because our institution’s subscription was no longer available. The reference lists of the articles included in the review, as well as those of published systematic reviews^5,6,8^ were examined to identify additional research studies. Citations were imported into EndNote 20®. After duplicates were removed, all references were screened by two independent reviewers based on the title and abstract. Articles that met the inclusion criteria, or were unclear regarding inclusion, were screened out, and the full text of each article was carefully reviewed before making a final decision. Another reviewer was consulted to clarify any discrepancies. Interrater agreement was measured using Cohen’s Kappa statistic (0.00-0.20 slight; 0.21-0.40 fair; 0.41-0.60 moderate; 0.61-0.80 substantial; 0.81-1.00 perfect agreement).^10^

### Data extraction and analysis

A reviewer collected information from included studies into a predefined Microsoft Excel spreadsheet, allowing for easy comparisons and reliable synthesis of results. The extracted data included patient characteristics (age, sex, lung function), details of the intervention, and means and standard deviations (SD) of baseline and follow-up measurements for each outcome of interest. In case of missing summary statistics, reviewers: i) attempted to derive the mean or SD based on available information (i.e., estimating the sample mean and SD from the sample size, median and/or interquartile range^11^) or extract data from graphs using WebPlotDigitizer; ii) contacted the study authors by email to request additional data; or iii) excluded the study from the meta-analysis. Mean change from baseline and SD were retrieved for each group using the MA-cont:pre/post effect size interactive tool^12^. The meta-analysis was conducted using R statistical software (version 4.2.0) with the metafor package,^13^ employing a random effects model. Results for the meta-analysis are displayed in forest plots. Two complementary methods were used to detect heterogeneity: visual inspection of the forest plot and numerical measures of heterogeneity (Cochran Q statistic and the inconsistency index (I^2^)).^14^ Heterogeneity was analysed with the I^2^ statistic and interpreted as low <30%, 30%≤ moderate <50%, 50% ≤ substantial <75% or high ≥75%.^15^ The mean change between groups for each outcome measure was compared with the established minimal clinically important difference (MCID).

### Risk of bias and quality of evidence assessment

Risk of bias was assessed using the Risk of Bias 2.0 tool.^16^ The Risk of Bias 2.0 tool assesses biases arising from the randomisation process (D1), deviations from the intended interventions (D2), missing outcome data (D3), measurement of the outcome (D4) and selection of the reported outcome (D5). Judgement can be ‘low’ or ‘high’ risk of bias or express ‘some concern’ and the overall risk of bias was reached using the signalling questions^16^. Risk of bias was assessed by one reviewer and checked by a second reviewer for accuracy. The Grading of Recommendations Assessment, Development, and Evaluation (GRADE)^17^ framework was used to assess the certainty of the evidence. Criteria for grading the level of certainty based on the GRADE guidelines are available in the supplementary material (Section A2). The GRADE assessment was performed separately by two independent reviewers, who then met to reach a consensus.

## Results

### Study characteristics

**Figure 1.**
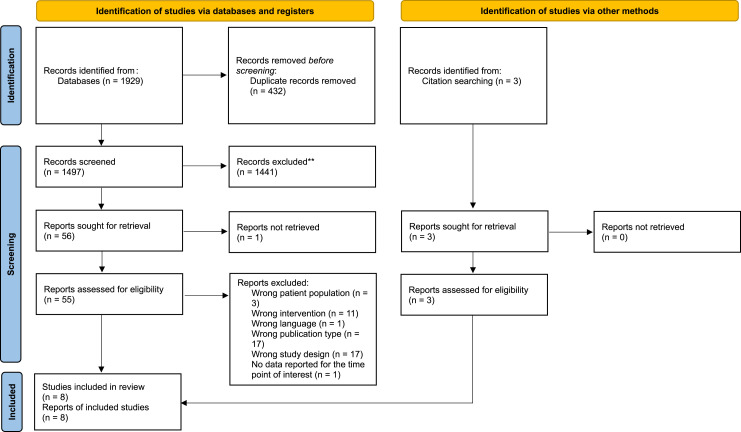
Flow diagram of studies included on the effects of pulmonary rehabilitation on the functional status (capacity and performance) of adults with interstitial lung disease according to the preferred reporting items for systematic reviews and meta-analysis (PRISMA).

Eight studies^18–25^ were included (Cohen’s Kappa = 0.61, substantial) and seven were considered for meta-analysis (Figure 1).

In total, 410 adults with ILD were included (mean age ranged from 59.1^25^ to 71.0^22^ years; mean forced vital capacity from 52.4^25^ to 76.0^24^ percentage predicted; diffusing capacity for carbon monoxide from 36.6^19^ to 59.4^23^ percentage predicted) (Table 1). Five studies^18,19,21–23^ included idiopathic pulmonary fibrosis (IPF) only, while three^20,24,25^ involved mixed ILD populations. Studies were conducted in outpatient (*n* = 7)^18,20–25^ or inpatient settings (*n* = 1)^19^. The duration of the PR programmes varied between 3^19^ and 26^20^ weeks, with sessions lasting between 30 min^24^ and 2 h.^22^ Inpatient PR sessions were conducted 5 to 6 times per week, while outpatient PR sessions were conducted 2 to 3 times per week. All studies included a combination of endurance and resistance training as well as education and/or psychosocial support. Educational sessions were conducted face-to-face; however, in seven studies, information was lacking regarding whether sessions were single or group-based, as well as details on delivery method, frequency, and/or duration of the educational component. Common topics of educational content were: nutrition,^18–20,22,24^ benefits of exercise and physical activity,^18,19,21,22,24^ anatomy and pathophysiology of chronic respiratory diseases,^18,22,24,25^ dyspnoea and symptom management,^18,21,22,24^ anxiety/depression/stress management,^18,20,22,24^ and the role and correct use of medication/oxygen therapy.^18,22,24^ All controls received usual care.

**Table 1.** Overview of the characteristics and results of the included studies assessing the effects of pulmonary rehabilitation on the functional status of adults with interstitial lung disease (*n* = 8).

Study ID, Country	Setting, Length	Participants		Pulmonary rehabilitation		Results	
		PR group	Control group	Exercise training	Education/psychosocial support	PR group	Control group
Dowman 2017, Australia^24^	Outpatient, 8 weeks	*N* = 74 Age = 69.0±11.0 Sex (M/F) = 44/30 FVC (%pred) = 76.0±18.0 DLCO (%pred) = 50.0±16.0	*N* = 68 Age = 70.0±11.0 Sex (M/F) = 43/25 FVC (%pred) = 75.0±20.0 DLCO (%pred) = 48.0±14.0	Frequency: 2x/week **Endurance training** Modality: Cycling (cycle ergometer); walking (treadmill or ground-based) Intensity: Cycling - 70% WR (6MWT)/symptom-limited (mBorg 3-4); walking - 80% 6MWD/symptom-limited (mBorg 3-4) Progression: Walking - increased by 0.25 km/h (treadmill) or 62m (corridor walking) weekly for speeds <0.3 km/h, or by 0.5 km/h or 125m for speeds ≥3 km/h; upon reaching max speed, reduced by 0.2-0.4 km/h and added 1%–2% gradient; subsequent sessions increased gradient by 1%–2%; cycling - intensity was increased by 15% weekly **Resistance training** Modality: Free weights (dumbbells) Load: 10-12 repetition-max/symptom-limited (Borg 12-14) Progression: Lower limb resistance exercises were increased every 2 weeks by 1-3 kg/symptom-limited (Borg 12-14); Upper limb resistance exercises increased by 2-5 repetitions until a total of 20 repetitions/symptom-limited (Borg 12-14) Time: 30 min Volume: 60 min/week	Frequency: - Duration: - Type: - Topics: Understanding lung disease, respiratory function tests, medications, home oxygen therapy, self-management including exacerbations, managing breathlessness, energy conservation, exercise and physical activity, stress and anxiety, nutrition and healthy eating, sexuality and intimacy, community resources, swallowing, continence, and airway clearance	6MWT, m Pre: 477.0±107.0 Post:m 500.7±107.0	6MWT, m Pre: 441.0±142.0 Post: 439.0±142.0
Gaunaurd 2014, United States^18^	Outpatient, 12 weeks	*N* = 11 Age = 71.0±6.0 Sex (M/F) = NR FVC (%pred) = 60.0±11.0 DLCO (%pred) = 44.0±11.0	*N* = 10 Age = 66.0±7.0 Sex (M/F) = NR FVC (%pred) = 61.0±14.0 DLCO (%pred) = 43.0±11.0	Frequency: 2x/week **Endurance training** Modality: Walking (treadmill); cycling (recumbent cycling) Intensity: 70%–80% HR max Progression: - **Resistance training** Modality: Elastic resistance bands Load: Elastic resistance bands (yellow); 2 sets of 10 repetitions Progression: Sets and repetitions increased to 3 × 15, with higher resistance (green bands) Time: 75 min Volume: 150 min/week	Frequency: - Duration: - Type: - Topic(s): Medication, breathing exercises, exercise training, nutrition, pulmonary physiology, and psychological coping strategies	IPAQ, MET-min Pre: - Post: - MD: 51.4±57.7	IPAQ, MET-min Pre: - Post: - MD: 20.9±37.2
Jackson 2014, United States^22^	Outpatient, 12 weeks	*N* = 11 Age = 71.0±6.0 Sex (M/F) = NR FVC (%pred) = 60.0±11.0 DLCO (%pred) = 44.0±11.0	*N* = 10 Age = 66.0±7.0 Sex (M/F) = NR FVC (%pred) = 61.0±14.0 DLCO (%pred) = 43.0±11.0	Frequency: 2x/week **Endurance training** Modality: Walking (treadmill); cycling (semi-recumbent cycling) Intensity: 60%–80% HR max Progression: - **Resistance training** Modality: Elastic resistance bands Load: Elastic resistance bands (yellow); 3 sets of 15 repetitions Progression: Increase in band resistance (yellow > red > green > blue) Time: 120 min Volume: 240 min/week	Frequency: Once every other week Duration: 15 min Type: Powerpoint presentations and handouts Topics: Medication use, breathing techniques, exercise strategies, proper nutrition, pulmonary physiology, and psychological coping mechanisms	6MWT, m Pre: 360.6±16.5 Post: 354.4±37.2	6MWT, m Pre: 339.4±34.4 Post: 324.1±35.5
Jarosch 2020, Germany^19^	Inpatient, 3 weeks	*N* = 34 Age = 68.0±9.0 Sex (M/F) = NR FVC (%pred) = 74.0±19.0 DLCO (%pred) = 44.1±15.4	*N* = 17Age = 65.0±10.0Sex (M/F) = NRFVC (%pred) = 72.0±20.0DLCO (%pred) = 36.6±18.8	Frequency: 5-6 days/week **Endurance training **Modality: Cycling (cycle ergometer), continuous or interval endurance training Intensity: 60%–100% WR max (CPET) – interval or continuous endurance training, if tolerated Progression: Symptom-limited (increase intensity/duration if mBorg <4) **Resistance training** Modality: - Load: 3 sets of 15-20 repetitions Progression: Symptom-limited (increase intensity/duration if mBorg <4) Time: - Volume: -	Frequency: - Duration: - Type: - Topics: Disease management, physical activity, nutritional counselling, and motivation	6MWT, m Pre: 415.0±101.0 Post: 469.0±139.5	6MWT, m Pre: 405.0±109.0 Post: 401.1±109.3
Nishiyama 2018, Japan^23^	Outpatient, 10 weeks	*N* = 13 Age = 68.1±8.9 Sex (M/F) = 9/6 FVC (%pred) = 66.1±13.2 DLCO (%pred) = 59.4±16.7	*N* = 15 Age = 64.5±9.1 Sex (M/F) = 12/1 FVC (%pred) = 68.7±19.5 DLCO (%pred) = 48.6±16.7	Frequency: 2x/week **Endurance training** Modality: Cycling (cycle ergometer); walking (treadmill) Intensity: Cycling -80% WR max (CPET); walking -80% 6MWD Progression: - **Resistance training** Modality: Elastic resistance bands Load: - Progression: - Time: - Volume: -	Frequency: - Duration: - Type: - Topics: -	6MWT, m Pre: 385.0±116.0 Post: 427.0±84.0	6MWT, m Pre: 476.0±128.0 Post: 472.0±130.0
Perez-bogerd 2018, Belgium^20^	Outpatient, 26 weeks	*N* = 30 Age = 64.0±13.0 Sex (M/F) = 22/8 FVC (%pred) = NR DLCO (%pred) = 41.0±13.0	*N* = 30 Age = 64.0±8.0 Sex (M/F) = 15/15 FVC (%pred) = NR DLCO (%pred) = 45.0±16.0	Frequency: 2-3x/week **Endurance training** Modality: Cycling (cycle ergometer); walking (treadmill); stair climbing Intensity: Cycling: 60% WR max (CPET); walking: 75% 6MWD; stair climbing: 2-min blocks (1-3 sets) Progression: Walking - 110% 6MWD; cycling - 85% WRmax; stair climbing - 3 sets of 2-min blocks(increments were symptom-limited until maximal targets reached) **Resistance training**Modality: Machine weights Load: 70% 1RM; 3 sets of 8 repetitions Progression: - Time: 90 min Volume: 270 min/week	Frequency: - Duration: 30 min Type: - Topics: -	6MWT, m Pre: 462.0±57.0 Post: 504.0±57.0	6MWT, m Pre: 491.0±57.0 Post: 474.0±57.0
						Steps/day, steps Pre: 5671.0±2598.0 Post: 5721.0±2598.0	Steps/day, steps Pre: 7013.0±2598.0 Post: 6593.0±2598.0
						Time spent in moderate PA Pre: 36.0 [25.0; 52.0] Post: 37.0 [26.0; 54.0]	Time spent in moderate PA Pre: 54.0 [38.0; 78.0] Post: 44.0 [31-0; 64.0]
Vainshelboim 2014, Israel^21^	Outpatient, 12 weeks	*N* = 15 Age = 68.8±6.0 Sex (M/F) = 10/5 FVC (%pred) = 66.1±14.8 DLCO (%pred) = 48.6±17.2	*N* = 17 Age = 66.0±9.0 Sex (M/F) = 11/6 FVC (%pred) = 70.1±17.4 DLCO (%pred) = 53.2±12.2	Frequency: 2x/week **Endurance training** Modality: Cycling (cycle ergometer); walking (treadmill or ground-based) Intensity: Cycling - 50%–60% WR max (CPET)/symptom-limited (mBorg 3-5); walking - 70%–80% 6MWD/symptom-limited (mBorg 3-5) Progression: Cycling - 60%–70% WR max (CPET); walking - 80%–90% 6MWD **Resistance training** Modality: Free weights (dumbbells) Load: Symptom-limited (mBorg 3-5); 1 set of 12-15 repetitions Progression: Symptom-limited (mBorg 4-6); 2 sets of 10-12 repetitions Time: 60 min Volume: 120 min/week	Frequency: - Duration: - Type: - Topics: Managing symptoms and motivation to be physically active	6MWT, m Pre: 513.0±108.0 Post: 583.4±108.0	6MWT, m Pre: 471.0±108.0 Post: 460.40±108.0
						30-s STS, reps Pre: 11.5±2.8 Post: - MD: 3.7±2.6	30-s STS, reps Pre: 13.7±4.5 Post: - MD: −0.4±2.5
Ku 2017, India^25^	Outpatient, 8 weeks	*N* = 20 Age = 59.1±10.4 Sex (M/F) = 8/12 FVC (%pred) = 52.4±13.7 DLCO (%pred) = NR	*N* = 20 Age = 62.1±14.5 Sex (M/F) = 8/12 FVC (%pred) = 58.6±13.7 DLCO (%pred) = NR	Frequency: 2x/week **Endurance training** Modality: Cycling (cycle ergometer); walking (not specified) Intensity: - Progression: - **Resistance training** Modality: Free weights Load: - Progression: - Time: 120 min Volume: 240 min/week	Frequency: - Duration: - Type: - Topics: Information about the disease, medications used in their treatment, how to change their behavior, and other non-drug treatments	6MWT, m Pre: 237.4±90.4 Post: 261.2±113.1	6MWT, m Pre: 208.0±93.7 Post: 211.4±108.8

Data are reported as mean ± SD and geometric mean [geometric 95% CI]. Legend: CPET, Cardiopulmonary exercise testing, DLCO, diffusing capacity of carbon monoxide, F, Female, FVC, Forced vital capacity, HR, Heart rate, IPF, idiopathic pulmonary fibrosis, IPAQ, International Physical Activity Questionnaire, MD, Difference in means, M, Male, mBorg, modified Borg scale, PA, Physical activity, PR, Pulmonary rehabilitation, WR, Work rate, 6MWT, Six-minute walk test, 1RM, One-repetition maximum, 30-s STS, 30-s sit-to-stand test.

### Effects of pulmonary rehabilitation on functional status

Seven studies evaluated functional capacity using the 6-min walk test (6MWT) (*n* = 7)^19^–^25^ and one of these studies also used the 30-s sit-to-stand test (30-s STS) (*n* = 1).^21^ Two studies assessed functional performance, measuring time spent in moderate PA (*n* = 1),^20^ and the number of steps per day with the SenseWear Armband (*n* = 1),^20^ and energy expenditure in MET-min using the international physical activity questionnaire (IPAQ) (*n* = 1)^18^.

**Figure 2.**
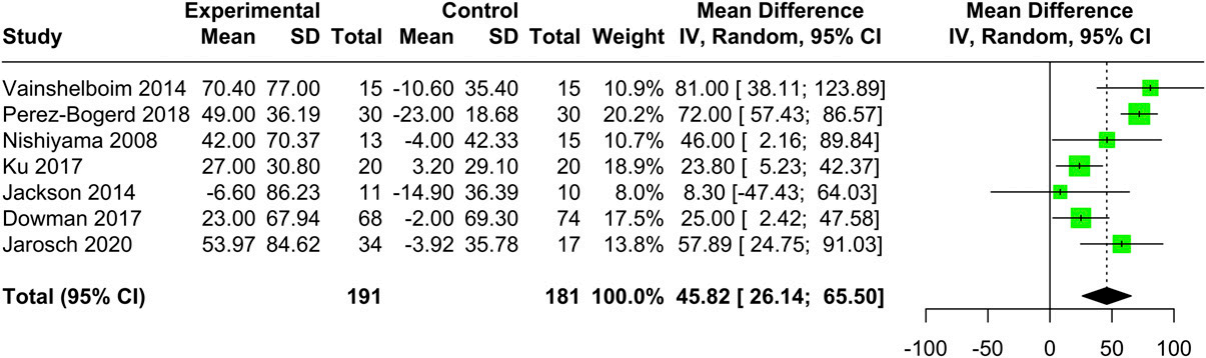
Meta-analysis of the effects of pulmonary rehabilitation versus control group on the six-minute walk test in adults with interstitial lung disease (*n* = 7). The point estimate and the statistical size (proportional area of the square) are shown. Horizontal lines indicate 95% confidence intervals. The pooled mean difference was calculated using a random effects model.

PR improved functional capacity measured with the 6MWT (MD 45.82 m, 95% CI [26.14; 65.50], I^2^ = 71.54%, *p* < .001) (Figure 2) compared to usual care, which exceeded the MCID (29-34m).^26,27^ The certainty level of evidence for the 6MWT was rated as low (Table 2). One study found a mean difference of 4.1 repetitions (PR: 3.7 ± 2.6 reps; control group: −0.4 ± 2.5 reps, *p* < .001) in the 30-s STS test^21^ in favour of the PR group, which is above the MCID of two repetitions.^28^ There were no improvements in steps/day^20^ or time spent in moderate PA^20^ compared to the control group; self-reported PA^18^ (measured with the IPAQ) improved significantly after PR (PR: 51.4 ± 57.7 MET-min; control group: 20.9 ± 37.2 MET-min, *p* = .03). Risk of bias assessment for each outcome measure per study is in supplementary material (Section A3 – Table S1).

**Table 2.** Grading of recommendations, assessment, development and evaluations (GRADE) of the effects of pulmonary rehabilitation on the six-minute walk test in adults with interstitial lung disease (*n* = 7).

Number of studies	Study design	Risk of bias	Inconsistency	Indirectness	Imprecision	Other considerations	Absolut effect (95% CI)	Certainty
Seven	RCTs	No serious limitations	Substantial heterogeneity^a^	Not detected	Inaccuracy detected^b^	-	45.82 m, 95% CI [26.14; 65.50], I^b^ = 71.54%, *p* < .001	Low (⊕⊕⊕⊘)

Data are presented as mean difference [95% CI]. Legend: CG, Control group, EG, Experimental group, RCTs, Randomised controlled trials.

^a^If the *p*-value of the Cochran Q test is < 0.1, and the Higgins I2 statistic is ≥ 50%, statistically significant heterogeneity is assumed – Rate down one level.

^b^For continuous results, information is likely to be insufficient if the total number of participants is less than 400 – Rate down one level.

## Discussion

This systematic review and meta-analysis demonstrated a positive effect of PR on functional capacity in people with ILD. Results for functional performance were inconclusive due to a lack of studies assessing this health domain.

The mean improvement in the 6MWT after PR was above the established MCID,^26^ suggesting that PR leads to clinically meaningful changes in functional capacity in adults with ILD, consistent with findings from a recent Cochrane review.^6^ However, substantial heterogeneity was found, likely attributable to differences in the duration and frequency of sessions, exercise modality, and training intensities. Functional tests that reflect activities other than walking and are applicable in space-constrained settings were hardly ever considered. In fact, only one study used the 30-s STS, and it found significant improvements above the MCID^28^ after PR. The inclusion of these tests is essential for a more comprehensive assessment of a person’s activity limitations.^3^

Functional performance was rarely assessed, and most measures were related to PA. The only reported improvement was in self-reported PA,^18^ but caution is warranted as this finding is based on just one study.^29^ A large heterogeneity and limited number of studies hampered both the summary of results and our ability to draw further conclusions on functional performance. Future studies should focus on functional performance to provide information on the impact of PR on patients’ ability to participate in activities related to self-care, housekeeping, and social and recreational activities.

This systematic review exclusively includes studies adhering to the PR definition, requiring both exercise training and education.^7^ Often, the impact of education on functional status outcomes is underestimated, with primary focus placed on the exercise component. However, awareness of the disease and its impacts, improved medication use (including oxygen therapy), and symptom management (such as energy conservation and dyspnea relief), along with other topics covered during the educational component, are likely to influence and optimise patients’ functional capacity and performance. Education can empower patients to better adapt to their environment and manage their condition,^30^ thereby promoting increased participation – effects that should not be dismissed. Nevertheless, there was often insufficient reporting on the education component, particularly concerning details such as session frequency and duration, delivery methods, session providers, and covered topics. These problems have also been observed in COPD studies and have been discussed elsewhere.^31^

The majority of included studies come from research on IPF, the most prevalent ILD.^32^ However, further research is needed for other ILDs, including exposure-related ILDs, connective tissue disease-associated ILDs, and fibrotic hypersensitivity pneumonitis. These conditions can lead to progressive pulmonary fibrosis,^33^ negatively impacting functional status. We acknowledge that only peer-reviewed publications and randomised controlled trials were included. Additional interventional studies may exist in the unpublished grey literature, and good-quality non-randomised studies might have complemented the results obtained.

## Conclusion

PR is effective at improving functional capacity and has been mostly measured with the 6MWT. Functional performance has been highly neglected in PR studies in adults with ILD, leading to limited and inconclusive findings. Future research should explore a wider range of activities and assess the effectiveness of PR in improving patients' daily lives, focusing on what they actually do rather than just what they are capable of doing.

## Supplemental Material

Supplemental Material - Functional status following pulmonary rehabilitation in people with interstitial lung disease: A systematic review and meta-analysis Supplemental Material for Functional status following pulmonary rehabilitation in people with interstitial lung disease: A systematic review and meta-analysis by Guilherme Rodrigues, Rute Santos, Rita Pinto, Ana Oliveira, and Alda Marques in Chronic Respiratory Disease
