# Supplementary material for: Functional status following pulmonary rehabilitation in people with interstitial lung disease: A systematic review and meta-analysis
Source: Chron Respir Dis. 2024 Oct 23;21:14799731241255138. doi: 10.1177/14799731241255138 (PMC11500222; doi:10.1177/14799731241255138)
Supplement: Supplemental Material - Functional status following pulmonary rehabilitation in people with interstitial lung disease: A systematic review and meta-analysis [file sj-pdf-1-crd-10.1177_14799731241255138.pdf]

Supplementary material

A1. Search Strategy

PubMed	Search string
#1	(Lung Diseases, Interstitial[MH] OR Interstitial Lung Disease[TIAB] OR Interstitial Lung Diseases[TIAB] OR Diffuse Parenchymal Lung Disease[TIAB] OR Diffuse Parenchymal Lung Diseases[TIAB] OR Interstitial Pneumonia[TIAB] OR Extrinsic Allergic Alveolitis[TIAB] OR Hypersensitivity Pneumonitis[TIAB] OR Pneumoconiosis[TIAB] OR Anthracosis[TIAB] OR Anthracosilicosis[TIAB] OR Asbestosis[TIAB] OR Berylliosis[TIAB] OR Byssinosis[TIAB] OR Caplan Syndrome[TIAB] OR Siderosis[TIAB] OR Silicosis[TIAB] OR Silicotuberculosis[TIAB] OR Anti-Glomerular Basement Membrane Disease[TIAB] OR Langerhans-Cell Histiocytosis[TIAB] OR Langerhans-Cell Granulomatosis[TIAB] OR Eosinophilic Granuloma[TIAB] OR Pulmonary Fibrosis[MH] OR Pulmonary Fibrosis[TIAB] OR Idiopathic Interstitial Pneumonias[TIAB] OR Cryptogenic Fibrosing Alveolitis[TIAB] OR Idiopathic Fibrosing Alveolitis[TIAB] OR Usual Interstitial Pneumonia[TIAB] OR Fibrocystic Pulmonary Dysplasia[TIAB] OR Nonspecific Interstitial Pneumonia[TIAB] OR Cryptogenic Organizing Pneumonia[TIAB] OR Bronchiolitis Obliterans Organizing Pneumonia[TIAB] OR Acute Interstitial Pneumonia[TIAB] OR Desquamative Interstitial Pneumonia[TIAB] OR Respiratory Bronchiolitis[TIAB] OR Lymphoid Interstitial Pneumonia[TIAB] OR Lymphocytic Interstitial Pneumonia[TIAB] OR Idiopathic Pleuroparenchymal Fibroelastosis[TIAB] OR Sarcoidosis[TIAB] OR Lymphangioleiomyomatosis[MH] OR Lymphangioleiomyomatosis[TIAB])
#2	(Lung Diseases, Interstitial[MH] OR Interstitial Lung Disease[TIAB] OR Interstitial Lung Diseases[TIAB])
#3	(Connective Tissue Diseases[MH] OR Connective Tissue Diseases[TIAB] OR Connective Tissue Disease[TIAB] OR Rheumatoid Arthritis[TIAB] OR Sjogren Syndrome[TIAB] OR Systemic Lupus Erythematosus[TIAB] OR Mixed Connective Tissue Disease[TIAB] OR Systemic Scleroderma[TIAB] OR Systemic Sclerosis[TIAB] OR Dermatomyositis[TIAB])

#4	2 AND 3
#5	1 OR 4
#6	(Rehabilitation[MH] OR Rehab*[TIAB] OR Exercise[MH] OR Exercis*[TIAB] OR Endurance Training[TIAB] OR Aerobic Training[TIAB] OR Resistance Training[TIAB] OR Strength Training[TIAB] OR Patient Education as Topic[MH:NOEXP] OR Education[TIAB] OR Self-Management[MH] OR Self-Management[TIAB] OR Social Support[MH] OR Social Support[TIAB] OR Psychosocial Support[TIAB] OR Psychological Support[TIAB])
#7	(Physical Fitness[MH] OR Physical Fitness[TIAB] OR Functional Performance[TIAB] OR Physical Performance[TIAB] OR Physical Function*[TIAB] OR Functional Status[TIAB] OR Activities of Daily Living[MH] OR Activities of Daily Living[TIAB] OR Activity of Daily Living[TIAB] OR Daily Activit*[TIAB] OR Physical Activity[TIAB] OR Sedentary Behavior[MH] OR Sedentary Behavior[TIAB] OR Sedentary Behaviour[TIAB] OR Sedentary Time[TIAB] OR Physical Inactivity[TIAB] OR Activity Monitor*[TIAB] OR Accelerometry[MH] OR Acceleromet*[TIAB] OR Actigraph*[TIAB] OR Pedometer[TIAB] OR Pedometers[TIAB] OR Step Count*[TIAB])
#8	5 AND 6 AND 7

Scopus	Search string
#1	TITLE-ABS-KEY (“Interstitial Lung Disease” OR “Interstitial Lung Diseases” OR “Diffuse Parenchymal Lung Disease” OR “Diffuse Parenchymal Lung Diseases” OR “Interstitial Pneumonia” OR “Extrinsic Allergic Alveolitis” OR “Hypersensitivity Pneumonitis” OR Pneumoconiosis OR Anthracosis OR Anthracosilicosis OR Asbestosis OR Berylliosis OR Byssinosis OR “Caplan Syndrome” OR Siderosis OR Silicosis OR Silicotuberculosis OR “Anti-Glomerular Basement Membrane Disease” OR “Langerhans-Cell Histiocytosis” OR “Langerhans-Cell Granulomatosis” OR “Eosinophilic Granuloma” OR “Pulmonary Fibrosis” OR “Idiopathic Interstitial Pneumonias” OR “Cryptogenic Fibrosing Alveolitis” OR “Idiopathic Fibrosing Alveolitis” OR “Usual Interstitial Pneumonia” OR “Fibrocystic Pulmonary Dysplasia” OR “Nonspecific Interstitial Pneumonia” OR “Cryptogenic

	Organizing Pneumonia" OR "Bronchiolitis Obliterans Organizing Pneumonia" OR "Acute Interstitial Pneumonia" OR "Desquamative Interstitial Pneumonia" OR "Respiratory Bronchiolitis" OR "Lymphoid Interstitial Pneumonia" OR "Lymphocytic Interstitial Pneumonia" OR "Idiopathic Pleuroparenchymal Fibroelastosis" OR Sarcoidosis OR Lymphangioleiomyomatosis)
#2	TITLE-ABS-KEY ("Interstitial Lung Disease" OR "Interstitial Lung Diseases")
#3	TITLE-ABS-KEY ("Connective Tissue Diseases" OR "Connective Tissue Disease" OR "Rheumatoid Arthritis" OR "Sjogren Syndrome" OR "Systemic Lupus Erythematosus" OR "Mixed Connective Tissue Disease" OR "Systemic Scleroderma" OR "Systemic Sclerosis" OR "Dermatomyositis")
#4	2 AND 3
#5	1 OR 4
#6	TITLE-ABS-KEY (Rehab* OR Exercis* OR "Endurance Training" OR "Aerobic Training" OR "Resistance Training" OR "Strength Training" OR Education OR "Self-Management" OR Education OR "Psychosocial Support" OR "Psychological Support")
#7	TITLE-ABS-KEY ("Physical Fitness" OR "Functional Performance" OR "Physical Performance" OR "Physical Function*" OR "Functional Status" OR "Activit* of Daily Living" OR "Daily Activit*" OR "Physical Activity" OR "Sedentary Behavio?r" OR "Sedentary Time" OR "Physical Inactivity" OR "Activity Monitor*" OR Acceleromet* OR Actigraph* OR Pedometer OR Pedometers OR "Step Count*")
#8	5 AND 6 AND 7

Web of Science Core Collection	Search string
#1	TS=(Interstitial Lung Disease OR Interstitial Lung Diseases OR Diffuse Parenchymal Lung Disease OR Diffuse Parenchymal Lung Diseases OR Interstitial Pneumonia OR Extrinsic Allergic Alveolitis OR Hypersensitivity Pneumonitis OR Pneumoconiosis OR Anthracosis OR Anthracosilicosis OR Asbestosis OR Berylliosis OR Byssinosis OR Caplan Syndrome OR Siderosis OR Silicosis OR Silicotuberculosis OR Anti-Glomerular Basement Membrane Disease OR Langerhans-

	Cell Histiocytosis OR Langerhans-Cell Granulomatosis OR Eosinophilic Granuloma OR Pulmonary Fibrosis OR Idiopathic Interstitial Pneumonias OR Cryptogenic Fibrosing Alveolitis OR Idiopathic Fibrosing Alveolitis OR Usual Interstitial Pneumonia OR Fibrocystic Pulmonary Dysplasia OR Nonspecific Interstitial Pneumonia OR Cryptogenic Organizing Pneumonia OR Bronchiolitis Obliterans Organizing Pneumonia OR Acute Interstitial Pneumonia OR Desquamative Interstitial Pneumonia OR Respiratory Bronchiolitis OR Lymphoid Interstitial Pneumonia OR Lymphocytic Interstitial Pneumonia OR Idiopathic Pleuroparenchymal Fibroelastosis OR Sarcoidosis OR Lymphangioleiomyomatosis)
#2	TS=(Interstitial Lung Disease OR Interstitial Lung Diseases)
#3	TS=(Connective Tissue Diseases OR Connective Tissue Disease OR Rheumatoid Arthritis OR Sjogren Syndrome OR Systemic Lupus Erythematosus OR Mixed Connective Tissue Disease OR Systemic Scleroderma OR Systemic Sclerosis OR Dermatomyositis)
#4	2 AND 3
#5	1 OR 4
#6	TS=(Rehab* OR Exercis* OR Endurance Training OR Aerobic Training OR Resistance Training OR Strength Training OR Education OR Psychosocial Support OR Psychological Support)
#7	TS=(Physical Fitness OR Functional Performance OR Physical Performance OR Physical Function* OR Functional Status OR Activit* of Daily Living OR Daily Activit* OR Physical Activity OR Sedentary Behavior OR Sedentary Time OR Physical Inactivity OR Activity Monitor* OR Acceleromet* OR Actigraph* OR Pedometer OR Pedometers OR Step Count*)
#8	5 AND 6 AND 7

A2. Criteria for grading the level of certainty based on the Grading of Recommendations, Assessment, Development, and Evaluations (GRADE) guidelines

1. Risk of Bias

- If 50-74% of included studies have an overall high risk of bias – Rate down one level.
- If 75% or more of the included studies have an overall high risk of bias – Rate down two levels.

2. Inconsistency
   - If the p-value of the Cochran Q test is <0.1, and the Higgins I^2 statistic is ≥50%, statistically significant heterogeneity is assumed – Rate down one level.
   - If only one of the criteria is present, examine whether the effect sizes of the studies are very different from each other and whether the 95% confidence intervals show minimal or no overlap, with a visual inspection of the forest plot, to confirm the presence/absence of significant heterogeneity (i.e., there is a significant inconsistency) – Rate down one level if detected.
3. Indirectness
   - If participants differ from those of interest – Rate down one level.
   - If the tested intervention in the included studies differs from the intervention of interest – Rate down one level.
   - If the outcomes (and outcome measures) differ from those of primary interest – Rate down one level.
   - If direct comparisons between two or more interventions of interest are not available (e.g., being interested in evaluating drug A vs drug B, but we only have studies that evaluate drug A vs placebo and drug B vs placebo) – Rate down one level.

   Note: Maximum rate down two levels.

4. Imprecision
   - Assess whether there is enough information to calculate an accurate effect estimate. For dichotomous results, information is likely to be insufficient if the total number of events is less than 300, or if the total (cumulative) sample size is less than the number of participants necessary for a study with adequate power (i.e., sample calculation). For continuous results, information is likely to be insufficient if the total number of participants is less than 400.
   - Assess the accuracy of the effect estimate (i.e., confidence interval)[4]. For dichotomous results, a confidence interval that includes a reduction in relative risk or an increase in relative risk greater than 25% in the limits of the confidence interval, suggests a decrease in the precision of the result. For continuous results, if the limits of the confidence interval cross the effect size (i.e., mean difference) of 0 in either direction suggests a decrease in the precision of the result.
   - Based on the above criteria, define whether there is any inaccuracy (rate down one level) or if there is a serious inaccuracy (rate down two levels).

If only one of the criteria is met, rate down one level; if the two criteria are met, rate down two levels.

5. Rating up the quality of evidence
   - We did not raise the level of evidence, as the grading of recommendations, assessment, development, and evaluations (GRADE) guidelines do not give clear guidance for rating up randomized controlled trials.

A3. Risk of bias assessment

Table S1: Risk of bias assessment (RoB 2 tool) of the functional status (capacity and performance) measures used in pulmonary rehabilitation of adults with interstitial lung disease.

Study ID	Outcome measure	D1	D2	D3	D4	D5	Overall
Dowman 2017[1]	6MWT	Low	Low	Low	Low	Some concerns	Some concerns
Gaunaurd 2014[2]	IPAQ	Some concerns	High	Low	High	Some concerns	Some concerns
Gaunaurd 2014[2]	6MWT	Some concerns	High	Low	Low	Some concerns	High
Jackson 2014[3]	6MWT	Some concerns	High	High	Low	Some concerns	High
Jarosch 2020[4]	6MWT	Some concerns	Some concerns	Low	Low	Some concerns	Some concerns
Nishiyama 2008[5]	6MWT	Some concerns	Some concerns	Low	Low	Some concerns	Some concerns
Perez-Bogerd 2018[6]	6MWT	Low	Some concerns	Low	Low	Some concerns	Some concerns
Perez-Bogerd 2018[6]	Steps/day	Low	Some concerns	Low	Low	Some concerns	Some concerns
Perez-Bogerd 2018[6]	Time spent in moderate PA	Low	Some concerns	Low	Low	Some concerns	Some concerns
Vanshelboim 2014[7]	6MWT	Low	Some concerns	Low	Low	High	High
Vanshelboim 2014[7]	30-second STS	Low	Some concerns	Low	Low	High	High
Ku 2017[8]	6MWT	Low	Some concerns	Low	Low	Some concerns	Some concerns

Abbreviations: D1, Deviations from the intended interventions; D2, Missing outcome data; D3, Measurement of the outcome; D4, Randomization process; D5, Selection of the reported result; IPAQ, International physical activity questionnaire; O, Overall; PA, Physical activity; 6MWT, Six-minute walk test; STS, sit-to-stand test

References

1. Dowman LM, McDonald CF, Hill CJ, et al. The evidence of benefits of exercise training in interstitial lung disease: a randomised controlled trial. *Thorax* 2017; 72: 610-619. 2017/02/19. DOI: 10.1136/thoraxjnl-2016-208638.
2. Gaunaurd IA, Gómez-Marín OW, Ramos CF, et al. Physical activity and quality of life improvements of patients with idiopathic pulmonary fibrosis completing a pulmonary rehabilitation program. *Respir Care* 2014; 59: 1872-1879. 2014/09/04. DOI: 10.4187/respcare.03180.
3. Jackson RM, Gómez-Marín OW, Ramos CF, et al. Exercise limitation in IPF patients: a randomized trial of pulmonary rehabilitation. *Lung* 2014; 192: 367-376. 2014/04/08. DOI: 10.1007/s00408-014-9566-9.
4. Jarosch I, Schneeberger T, Gloeckl R, et al. Short-Term Effects of Comprehensive Pulmonary Rehabilitation and its Maintenance in Patients with Idiopathic Pulmonary Fibrosis: A Randomized Controlled Trial. *J Clin Med* 2020; 9 2020/05/28. DOI: 10.3390/jcm9051567.
5. Nishiyama O, Kondoh Y, Kimura T, et al. Effects of pulmonary rehabilitation in patients with idiopathic pulmonary fibrosis. *Respirology* 2008; 13: 394-399. 2008/04/11. DOI: 10.1111/j.1440-1843.2007.01205.x.
6. Perez-Bogerd S, Wuyts W, Barbier V, et al. Short and long-term effects of pulmonary rehabilitation in interstitial lung diseases: a randomised controlled trial. *Respir Res* 2018; 19: 182. 2018/09/22. DOI: 10.1186/s12931-018-0884-y.
7. Vainshelboim B, Oliveira J, Yehoshua L, et al. Exercise training-based pulmonary rehabilitation program is clinically beneficial for idiopathic pulmonary fibrosis. *Respiration* 2014; 88: 378-388. 2014/10/25. DOI: 10.1159/000367899.
8. Vivek KU, Janmeja AK, Aggarwal D, et al. Pulmonary Rehabilitation in Patients with Interstitial Lung Diseases in an Outpatient Setting: A Randomised Controlled Trial. *Indian J Chest Dis Allied Sci* 2017; 59: 75-80.
